# Unpacking the Cinderella black box of complex intervention development through the Partners at Care Transitions (PACT) programme of research

**DOI:** 10.1111/hex.13682

**Published:** 2023-04-25

**Authors:** Jenni Murray, Ruth Baxter, Rebecca Lawton, Natasha Hardicre, Rosie Shannon, Joseph Langley, Rebecca Partridge, Sally Moore, Jane K. O'Hara

**Affiliations:** ^1^ Yorkshire Quality and Safety Research Group Bradford Institute for Health Research Bradford West Yorkshire UK; ^2^ School of Psychology University of Leeds Leeds UK; ^3^ Lab4Living Sheffield Hallam University Sheffield UK; ^4^ School of Healthcare University of Leeds Leeds UK; ^5^ Present address: School of Psychology University of Leeds Leeds LS2 9JT UK; ^6^ Present address: Leeds Beckett University & Leeds Academic Health Partnership University of Leeds Worsley Building Leeds LS2 9LU UK; ^7^ Present address: Academic Unit of Elderly Care Research Bradford Institute for Health Research Temple Bank House, Bradford Royal Infirmary, Duckworth Lane Bradford West Yorkshire, BD9 6RJ UK

**Keywords:** complex intervention development, transitions of care, older people

## Abstract

**Introduction:**

Complex intervention development has been described as the ‘Cinderella’ black box in health services research. Greater transparency in the intervention development process is urgently needed to help reduce research waste.

**Methods:**

We applied a new consensus‐based framework for complex intervention development to our programme of research, in which we developed an intervention to improve the safety and experience of care transitions for older people. Through this process, we aimed to reflect on the framework's utility for intervention development and identify any important gaps within it to support its continued development.

**Findings:**

The framework was a useful tool for transparent reporting of the process of complex intervention development. We identified potential ‘action’ gaps in the framework including ‘consolidation of evidence’ and ‘development of principles’ that could bracket and steer decision‐making in the process.

**Conclusions:**

We consider that the level of transparency demonstrated in this report, aided through use of the framework, is essential in the quest for reducing research waste.

**Patient or Public Contribution:**

We have involved our dedicated patient and public involvement group in all work packages of this programme of research. Specifically, they attended and contributed to co‐design workshops and contributed to finalizing the intervention for the pilot evaluation. Staff also participated by attending co‐design workshops, helping us to prioritize content ideas for the intervention and supporting the development of intervention components outside of the workshops.

## 1 INTRODUCTION

Literature describing complex intervention development has lagged behind evaluation and implementation sciences and has been described as the ‘Cinderella’ black box in health services research.^1^ Despite the publication of guidance and frameworks on the development of complex interventions over many years,^2^, ^3^, ^4^, ^5^, ^6^ replicable and detailed reporting of the collective actions taken during the development of complex healthcare interventions is somewhat limited. Reasons for this lag are likely multifactorial and interlinked, including, for example, lack of evidence linking use of intervention development guidance frameworks to positive outcomes^7^; a lack of clarity about which actions within the guidance contribute to clinically effective interventions^8^, ^9^, ^10^; adoption of varying and uncertain approaches^11^ and a lack of welcome ‘space’ (through journals) to host ‘messy’ research.^1^ From our own observations, we would add to that list: an unchallenged acceptance that snapshots of, for example, co‐design steps sufficiently represent the main process of development; a scientific narrative that has predominantly focused on evaluation, specifically randomised controlled trials (RCT) and an underappreciation of the imperative to broaden the narrative and share the learning to progress the science on complex intervention development. Research waste (i.e., research that is, among other things, poorly designed or unnecessary) is of ongoing concern,^12^, ^13^ and it is thought that greater transparency about the intervention development process will support the learning needed to shift trends from negative and inconclusive trials towards more positive outcomes.^7^, ^14^

Recently, O'Cathain et al.^14^ published a consensus‐based framework to guide decision‐making in complex intervention development. This framework lends itself to more systematic and transparent reporting.^15^ The framework suggests a set of 11 key actions and 5 principles, collectively forming a logic model. The actions include planning the process through to intervention refinement (Table 1). The principles include being dynamic, iterative, creative, open to change and looking towards evaluation (Table 2). The framework is critical for the field of complex intervention development in that it both acknowledges the challenges and nuances of the process and grants permission to researchers to be open about these. There is now an opportunity to start unpacking this major black box within health services research, to contribute to the emerging science on complex healthcare intervention development and to support efforts to reduce research waste.

**Table 1:** The three phases of intervention development in the partners at care transitions (PACT) study and how these align with O'Cathain et al.'s^14^ recommended complex intervention framework actions

Existing actions in framework	PACT Phase 1 (early preparation work)	PACT Phase 2 (gathering the evidence to develop a theory of change)	PACT Phase 3 (active phase of intervention design)
I Planning	We explored a range of literature to confirm the views of stakeholders (see Action II below) and identified transitions for older people to be high on UK policy agenda. We defined a set of principles to guide intervention development. Based on the funder's request, we suggested potential intervention components and the care settings for them.	Planning for and within each WP was a constant part of the research programme.	Planning for and within each WP was a constant part of the research programme.
II Stakeholder Involvement	The main problem to be addressed and the target population were originally identified by a panel of local community patients. They felt that people want to participate more in their care but had concerns about taking on ‘responsibility’ for their own safety. They identified the main outcomes to be patient experiences and safety.	We established a dedicated panel of comprising older people who regularly contributed to and influenced work in every WP. Initially, we worked with the panel to explore care experiences and to help them understand the concept of involvement. We held two stakeholder workshops with hospital and community staff, patients and academics to explore their views on what was important and feasible to address in transitions and what the intervention might include.	The intervention development group (comprising patients, carers, hospital and community staff) offered lots of suggestions and encouraged us to explore better ways to communicate the concept of involvement.
III Establish a team	The research team included an operational team of four researchers with the Chief Investigator and work package leads from the PMG. Team members were new to each other so needed to learn about each other's working styles.	As the study moved towards active intervention development, the team were increasingly supported by experienced research nurses providing a future focus on service context.	The team expanded to include two intervention designers with expertise in co‐design. Before and between co‐design workshops, the operational and design team met to plan, create a shared language and understanding of our programme theory and project boundaries and to develop the intervention ideas into a prototype.
IV Review published research evidence	The original proposal outlines the policy context, the evidence in relation to transitions and related interventions and opportunities for improvement from a theoretical perspective.	Gaps in the evidence base were identified in relation to both understanding the concept of patient involvement in care and in ascertaining the active ingredients of existing transitions interventions. A systematic review of patient involvement clarified this concept,^16^ whilst a review of reviews of transitions intervention provided little clarity on the active components.	More broadly, the evidence that we gathered included literature reviews, scoping reviews, evidence from our own WPs, stakeholder events and patient panel feedback throughout the programme.
V Drawing on existing theories	Our proposal outlined our plans to apply a novel theoretical approach to transitional care. This shifted the focus from a traditional safety management approach with control and standardization of work (with fixed intervention components) to a resilience‐based approach allowing for flexibility according to system requirements.^17^	In consolidating the evidence from our first two work packages, we applied FRAM,^18^ which allowed us to fully explore the transitional system components as functions.^19^	We modified our original principles and identified functional activities of intervention from WP1 and 2, as part of WP4 involving stakeholder engagement.
VI Develop programme theory		Application of the FRAM allowed us identify the patient pathway that connected risk management strategies in hospital and the activities that patients undertake at home and their involvement in these during the hospital stay. This clear pathway allows us to gauge how our intervention would create change.	
VII Primary data collection		WP1 and WP2 studies^20^, ^21^, ^22^, ^23^ were the primary forms of data collection informing the subsequent stages.	
VIII Understand the context		Through WP1 and WP2, we developed a comprehensive understanding of patient and staff experiences of receiving and delivering care during transitions from hospital to home. We conducted a scoping review of innovations being used in the NHS to explore which formats might be more readily adopted in practice.	We consulted with NHS and social care staff with experience of supporting older people and who had an in‐depth understanding of the systems in which the intervention would be delivered. Further exploration of the context in which the intervention would be delivered would only happen during subsequent pilot and feasibility phases.
IX Attention to future implementation	Creation of a principle to develop an intervention based on functional aims allowing for flexibility in implementation. Focusing on a single component intervention that would require minimal additional resource alongside more effective use of existing resources would therefore minimize future additional costs to facilitate future implementation.		Consultation with NHS staff during development of content and format of intervention about what was already being delivered, the challenges and opportunities for implementation, likely effectiveness and resource burden. We explored barriers and facilitators to delivering the fixed components within the intervention through our scoping review and through contact with local services.
X Design/refine intervention		Co‐design workshops, additional PPI sessions and consolidation of evidence external to the workshops.	This was future work incorporated into our pilot and feasibility studies

Abbreviations: PACT, Partners at Care Transitions; PMG, Programme Management Group; WP, work package.

**Table 2:** Summary of how the PACT programme of research addresse the principles of the O'Cathain et al.^14^ framework

Framework principles	How each priniciple was addressed in the PACT research programme
Dynamic	An ambitious programme of research with the first three work packages overlapping and informing each other. Subsequent work packages were sequential, with rapid turnaround of results to feed into the next stage.
Iterative	One formal iteration of the intervention planned within WP4 with opportunity to understand some of the requirements for implementation. Opportunities to refine the intervention and further develop the implementation package were available during feasibility testing.
Creative	Use of Safety II theory^17^ as an underpinning concept for intervention development. Fully exploring the meaning of patient involvement. Using FRAM^18^ to consolidate our evidence and inform our theory of change and approaching transitions interventions in a novel way.
Open to change	An original planned review on older people's experiences of transitions was replaced (because of the identification of an existing recent review) with a review on how patients enacted involvement.^16^ The creation of an intervention that was, to some extent, different to that envisaged at the outset including the intended location for delivery. Going forward, being open to feedback from pilot and feasibility testing requiring modification to the intervention.
Looking forward to evaluation	Adopting from the outset an approach that focused on building an intervention based on functions rather than components to allow for some local adaptation.

Abbreviations: FRAM, Functional Resonance Analysis Method; PACT, Partners at Care Transitions.

Here, we report the process of developing a complex healthcare intervention to improve the safety and experience of older people (aged 75 years and older) transitioning from hospital to home (Table 3) and the extent to which the steps that we took address the actions within the reported framework. In line with O'Cathain et al.'s^14^ recommendations, we report the work that preceded, and was fully embedded in, the active designing of the intervention itself. Throughout, we highlight the challenges encountered and the nuanced decisions that were made in line with enacting the principles of complex intervention development. Finally, we explore important gaps within the framework and offer suggestions for its enhancement.

**Table 3:** Outline of work packages (WP) within the PACT research programme

Overarching aim	To improve the safety and experience of care transitions for older people
WP1	Qualitative study exploring the experiences and involvement of patients and carers during care transitions.^20^, ^21^
WP2	Qualitative study exploring the factors that facilitate successful transitions of care within high‐performing (positively deviant) teams across primary, secondary and community care, and the ways in which staff overcome the challenges faced in their everyday work.^22^, ^23^
WP3	Developing a measure (PACT‐M) to assess the quality and experience of transitions for older people. This measure was to be considered as a secondary outcome in the full cluster RCT. Not described in the current paper as this work was not central to intervention development.^24^, ^25^
WP4	Co‐design the PACT intervention and perform a formative evaluation of the prototype intervention.^26^
WP5	Testing the feasibility of delivering the new PACT intervention within a cluster RCT with a nested qualitative evaluation to inform the implementation strategy.^27^
WP6	Cluster RCTto assess the effectiveness and cost‐effectiveness of the PACT intervention.

Abbreviation: PACT, Partners at Care Transitions.

## 2 THE PROCESS OF INTERVENTION DEVELOPMENT

**Figure 1:**
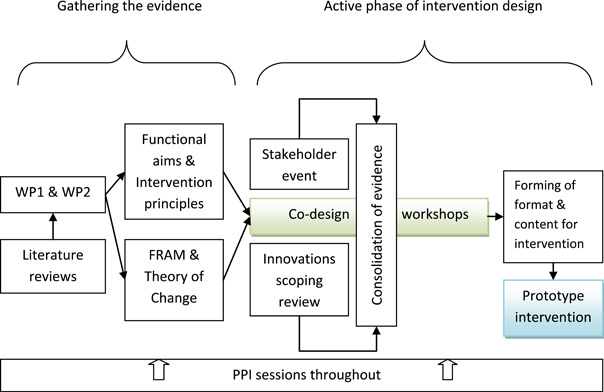
Main steps in intervention development

The main steps undertaken in developing the intervention are shown in Figure 1. Each of the three phases is described below.

### 2.1 Phase 1: Early preparatory work

Transitional care for older people was raised as a concern by a local quality and safety stakeholder group. The group, which represents a diverse local community, comprises 25 local community members. At this stage, they had been working regularly with the research team, based at the local NHS Trust, for 2 years. Work to maintain the group and its diversity through connecting with local community groups continues to this day through dedicated patient and public involvement (PPI) leads connected with the research team. Amongst other things, the group's role involves highlighting priorities for research and one of these areas was patient participation in care with an expressed concern for the safety of older people. On exploring the literature, we learned that the transitional period from hospital to home is a risky time for older people, exacerbated by reduced lengths of stay and ongoing complex care needs.^28^, ^29^ Through discussions with the group and exploration of the literature, we learned that greater patient involvement in care was desirable and potentially amenable to change^30^, ^31^, ^32^ but that there was some reluctance to adopt specific activities as part of a safety role,^33^, ^34^, ^35^, ^36^ for example, when challenging staff or monitoring whether particular practices occur.^34^, ^35^, ^36^ These findings led us to prepare a research proposal that, in line with emerging safety science, was underpinned by a Safety II approach.^17^ With this, there is a shift towards understanding everyday performance and strengthening the system to enhance the number of things that go right. This is opposed to the traditional approach that exclusively focuses on, and seeks to prevent, adverse events (now termed Safety I).^17^ For the purposes of this programme, the aim was to create opportunities to support patients and staff to respond to, monitor, learn from and anticipate potential threats.^17^

On application, funders required us to outline the format of the intervention. Based on existing evidence, we anticipated that we would design a patient‐owned record and a predischarge meeting delivered in the hospital and a postdischarge telephone call to patients. Documenting this requirement here is important as it allows readers to consider how we have responded to the framework principle of being ‘open to change’ (see Table 4). The effectiveness of the trial of the intervention also required an important and readily measurable patient safety outcome and reviewers advised targeting emergency readmissions as the primary outcome.

**Table 4:** Early and revised intervention principles guiding intervention development

Early intervention principles	Revised intervention principles (before the active intervention development phase)
To help patients/carers better navigate and manage the transition from hospital to home in partnership with formal carers. Doing this *with* patients and those caring for them. Being flexible so fitting within different systems of care.	Creating resources that enable patients/carers to reach in to the system such that they can practice for going home. These resources will support patients to (1) ask questions and (2) actively ‘do care’. Supporting patients/carers to be knowledge brokers, making themselves visible to the system, reducing safety gaps. Making the care system more visible to support patients/carers to better navigate and interrogate it. Creating a gentle scaffold to systematize patients reaching in. This will be in the form of reciprocal staff/service involvement work through staff facing flexible intervention components.

At the outset of the study, we defined our own set of guiding principles for intervention development (see Table 4). One such principle (to develop a flexible intervention that was adaptable to different care systems) demonstrates early consideration to future implementation, in line with existing evidence^37^ (see Action IX in Table 3). The need for flexibility additionally guided us to develop an intervention around functional aims rather than purely fixed components.^38^, ^39^, ^40^

### 2.2 Phase 2: Gathering the evidence to develop a theory of change

Four main evidence‐gathering activities that contributed to informing intervention development included two literature reviews (a systematic review on patient involvement in care and a review of reviews on the transitions intervention literature) and primary data collection (WPs 1 and 2) (see Actions IV and VII in Table 1). The literature reviews clarified unchallenged assumptions about the meaning of patient involvement and its enactment in a hospital setting^16^ and showed that multicomponent and often condition‐specific transitions interventions articulated patient involvement as patient education or self‐management that differed from how we conceived patient involvement, as a nuanced and dynamic interactional process. This factor indicated our approach to be novel, with the intervention literature offering limited guidance.

Numerous early team discussions, including regular PPI, enabled us to start exploring how patient involvement in hospital for this population could happen in healthcare. We observed the role of patients in plugging gaps in care delivery and error checking as a way of supporting system resilience^41^, ^42^ but noted that some of these safety activities necessitated patients having to express concerns to staff. This was considered to be particularly challenging for many patients, particularly older, non‐English‐speaking or more unwell patients.^43^, ^44^, ^45^ Additional challenges to enhancing patient involvement became apparent through our early work within the study (see Action II in Table 1). These included, for example, staff pressures that prioritize rapid discharges over planning for a transition, risk ‘management’ practices in hospital that shifted risk to patients after discharge,^23^ care processes that are often not seen by patients and carers and patients' capacity or preferences for involvement in care.^21^ We reflected on concerns that an intervention targeting patient involvement to close system gaps may represent a ‘sticking plaster’ and that the onus of responsibility for enacting care should not be placed unduly on vulnerable patients. Our discussions also helped us to clarify the basis for what would be our theory of change. Specifically, we understood that patients needed to ‘reach in’ to a system *and* that staff within the system needed to ‘reach out’ to patients but that critically, many interacting contextual factors would make this unlikely without the support and without changing mindsets.^41^ This new understanding fitted with our considered original plans of having patient‐facing core intervention components to support involvement, scaffolded by flexible staff‐facing components.

To establish key areas to target for our intervention, to guide the delivery location and to inform our theory of change, we mapped data gathered from work packages 1 and 2 (specifically in relation to care activities) using the Functional Resonance Analysis Method (FRAM).^18^ We identified four key functional activities that patients undertake after discharge: managing medicines; managing health and well‐being; managing activities of daily living and appropriately escalating care to acute hospitals.^19^ Our theory of change suggested that the ability of patients to carry out these activities successfully would be partly determined by the extent of their involvement in preparing for these during the hospital stay^19^ (see Action VI in Table 1). Consequently, our intervention needed to support patient involvement in targeted specific hospital‐based activities to improve outcomes after discharge. Locating *delivery* of the intervention in the acute setting represented a change from our original plans that envisaged delivery of an intervention split between secondary and primary care. We did, however, envisage that the intervention could be carried through to the community by patients, akin to the ‘red book’ used in UK maternity, health visiting and community services.

### 2.3 Phase 3: Active phase of intervention design

Having clarified our theory of change, we revisited our earlier intervention principles to provide a more prescriptive guide for the active phase of intervention design (Table 4). We also created statements of functional aims for each of our five functional activities (Table 5). Both the intervention principles and functional aims were important benchmarks for judging potential content and format ideas to be taken forward for the intervention.

**Table 5:** Functional activities undertaken by patients after discharge from hospital and corresponding functional aims of the intervention

Functional activities	Functional aims of intervention^a^
Understanding and managing medicines (this is a combination of two activities: managing TTO^b^ medicines and managing ongoing medicines)	To resource patients to correctly take their medications^1^, effectively transition between TTOs^b^ and their ongoing medications^2^ and to identify and overcome any medication problems^3^ that they may encounter.
Understanding and managing health and well‐being	To resource patients to understand their health and well‐being and the care therein^1^, their role in facilitating recovery^2^ and to be ready to resume responsibility^3^ (as appropriate) for their health and well‐being
Managing activities of daily living	To resource patients to retain their autonomy and minimize the effects of deconditioning.
Knowing what to expect and how to escalate care appropriately after discharge.	To resource patients to seek help in an appropriate and timely manner for when any aspect of their health and well‐being deteriorates acutely.

^a^ Numbers 1 to 3 in the functional aims represent subaims.

^b^ TTO—to take out medicines.

Four key overlapping but distinct activities during the active phase of intervention development occurred throughout 2018 (Figure 1) and included co‐design workshops; consolidation of evidence; formation of content and format for the intervention and the prototype. These are described below.

#### 2.3.1 Co‐design workshops

We held four 3‐h co‐design workshops facilitated by intervention design experts. Attendees at the workshops included PPI members, hospital‐based staff (pharmacy, discharge co‐ordinators, geriatricians), community social care and primary care staff and a representative from Healthwatch England. Each workshop in turn aimed to (1) explore personal experiences of transitions; (2) use patient stories to explore facilitators to involvement; (3) develop content and format ideas for the intervention and (4) review the prototype. The outputs from the last two workshops are described below in the sections on forming the content and format for the intervention and reviewing the prototype.

Challenges to the co‐design process and our responses to these included the following:

(1) The first two workshops were designed to orientate participants using visual aids and creative approaches such as LEGO® Serious Play® and Persona's to attune them to our intentions for the intervention. This included ensuring that they understood the boundaries of the research in relation to the intervention principles (Table 4) and functional aims (Table 5). This meant that inputs into the content and format of the intervention only took place during the last two workshops. To enhance opportunities for greater input, we continued to hold our usual PPI meetings in between workshops. For example, in workshop 2, we used a visual aid to facilitate discussions on how participants prepared for returning home after a holiday. This was designed to encourage them to think about how they were involved in planning. After this workshop, we repeated this process with our PPI group, but the visual aid substituted returning home from holiday with returning home from hospital, enabling the group to explore how their planning skills could be applied to a hospital setting (albeit with caveats). Holding these separate PPI sessions was valuable to our PPI members, most of whom were aged 75 years or older and with existing health conditions, which made participation in the larger co‐design group workshops more challenging. Further, these sessions allowed us to explore issues in more depth.
(2) Having the theory of change based on robust and thorough evidence and a clear set of intervention principles meant that we had clear boundaries to guide workshop participants. We made these intervention principles clear to the group during the second workshop. Despite this, some participants found balancing the intervention principles very challenging and often promoted ideas (based on their own experiences) that did not always meet with our principles or theory of change. These included format suggestions like wrist bands to identify vulnerability, personal dignity boxes, creating new wards and new community‐based initiatives.
(3) The workshops were spaced at 2‐monthly intervals. Therefore, participants needed reorientating at the beginning of each workshop to encourage relevant suggestions for the intervention.

Despite these challenges, workshop participants contributed to the development of the intervention with ideas such as having a plan for the day; an advocate; a ward induction; medication checklists and records at the bottom of the bed that patients could read. More content‐based suggestions related to knowing reasons for admission and change in medications, knowing what was to be expected of the patient on the ward and having permission to do various things on the ward including moving around or leaving the ward for access, for example, hospital green space.

#### 2.3.2 Consolidation of evidence to inform the content and format for the intervention

Between the second and the fourth workshops, the PACT team undertook intensive consolidating work, bringing together the various sources of evidence to streamline the content and format ideas in preparation for the final workshop. Sources of evidence included the co‐design workshops, a scoping review of grey literature on service improvement innovations for transitions for older people and a PACT transitions stakeholder event held in June 2018 and attended by 26 health and social care staff. Content ideas from the scoping review were cross‐checked with the findings from WP1 and WP2 to ensure representativeness. For the scoping review (conducted between December 2017 and May 2018), the websites of 42 organizations were explored to identify any relevant patient‐facing innovations. Keywords searched included, for example, older people, discharge, transitions, handover and involvement. We recorded the nature of potential innovations of interest including their functions and downloaded any material relevant to the innovation. Rather than inform decision‐making about what components to include in the intervention, this scoping review was pre‐emptive work intended to provide ‘off the shelf’ intervention components, should they be required. It also enabled us to understand what formats were deliverable in everyday practice (see Action VIII in Table 1).

#### 2.3.3 Formation of intervention content and format for the intervention

From the evidenced sources described above, we amassed a list of 62 individual content suggestions (directed towards patients, carers and staff) that we mapped to each of the four functions. Each suggestion was rated as weak, moderate or strong according to the extent to which it (a) met the key aims of that function; (b) supported patient involvement; (c) added value (i.e., it was viewed as new, doable and would contribute to better patient outcomes) and (d) supported system resilience. To enable us to move at pace, we consulted six staff (general practitioner, Consultant in Elderly Care, nurses and a pharmacist) and the patient panel from the co‐design group using a consensus approach (Table 6). We settled on 31 content items to take forward.

**Table 6:** Examples of content suggestions and decision‐making on inclusion/exclusion from intervention

Suggestion^a^	Functional activity	Did it meet the stated functional aim?	Did it support system resilience?	Consultation^b^ conclusion
Patients keep their personal belongings safe, for example, glasses dentures, hearing aids	Managing activities of daily living	To some extent	Indirectly	Important but difficult to resource. Excluded.
Patients communicate to staff what their care needs are	Understanding and managing health and well‐being	To some extent	Indirectly	Not taken forward to consultation as already done in practice.
Patients are empowered to ask questions about their medications and know who to ask/contact	Understanding and managing medicines	Yes	Yes	All agreed important and deliverable. Included.
Patients know what ‘side effects’ might be normal after discharge	Knowing what to expect and how to escalate care appropriately after discharge	Yes	Indirectly	All agreed important and deliverable. Included.

^a^ From a range of sources (co‐design group, scoping review, stakeholder event, WP1 and 2).

^b^ Consultations were with two clinicians, a health psychologist, three nurses and the PPI panel.

Format suggestions from both the workshops and the scoping review were submitted to the design team, alongside the final content list. Repeated exchanges between the design team and the PACT team ensured alignment in thinking. Initial ideas for the format that were taken forward for discussion included a question card, an about me card, a leaflet, a red bag, passports, a communication board and video and lunch time and locker mats. Numerous discussions within the co‐design workshops and between the PACT team and the design team took place about how to take forward format ideas related to cost, the extent to which it supported patient involvement and met with funders' aims in terms of scope, the extent to which it could meet all of the intended functional aims, practical implementation (including the capacity of staff to deliver the intervention and generalizability) and infection control. For the purposes of developing a prototype, we were primarily concerned with taking forward suggestions that would support patients to ‘reach in’ and support system resilience.

#### 2.3.4 Reviewing the prototype

We created the ‘Getting Home, Staying Home’ intervention, which comprised a patient‐held booklet, a ward induction leaflet, a stand‐up question card and a ‘your hospital record’ sheet and a patient‐friendly discharge letter. These were housed in a purpose‐designed envelope. The branding included coloured icons depicting each function and appeared on each component in the intervention pack. Our initial idea at the grant proposal stage was for a patient‐owned record supported by a predischarge meeting and a postdischarge telephone call to patients. Our evidence indicated that a predischarge meeting, as a one‐off activity, would not support sustained patient involvement to adequately prepare for the postdischarge period. Further, at the time, there was little evidence from the transitions intervention literature that a postdischarge telephone call would impact on patient experience, safety and hospital readmissions and that with limited resources, this was unlikely to be implementable. We considered that the booklet, stand‐up question card and hospital record sheet could be used to encourage communication between staff and patients as an ongoing activity to support and encourage continued involvement in the hospital. Both the booklet and the induction leaflet were seen to be important in increasing the visibility of ward processes, allowing patients and carers to know more about their care and therefore enable greater participation in and preparation for the transitional period. The template patient‐friendly discharge letter (which was developed outside the workshops) was seen as a way to support patients to navigate through postdischarge care. At the final co‐design workshop, participants reviewed the patient‐held booklet, question card and hospital record. Their comments on the usefulness, usability, length (wordiness), wording, order, branding and size (of font and booklet) were incorporated into reworking the booklet. They also suggested a video version of the booklet that we took forward for future development. We used personas to engage the workshop participants in discussions about how patients and staff might engage with the booklet. Various implementation ideas were also suggested during the workshop and discussed within the group and these included posters, staff training, stickers, lanyards, electronic alerts, wrist bands and the use of safety huddles to promote the intervention. The timing of delivering the intervention to patients within the hospital stay was also discussed. The prototype intervention was taken forward to a formative evaluation to explore its initial acceptability, to learn about its implementation in practice and to inform further development. At this stage, we had not fully explored how staff could respond flexibly to the intervention functions to scaffold the intervention and enhance patients reaching in. We envisaged that this would be explored during the pilot and trial feasibility studies.

## 3 DISCUSSION

We have developed a novel transitions intervention for older people based on theoretical principles of resilient healthcare^17^ and patient involvement.^16^ This intervention aims to support patients to reach into the system during their hospital stay, to ‘know more’ and ‘do more’ in preparation for managing their own care and well‐being on returning home. This is a departure from existing transitions interventions that purport the need for multiple and contrasting fixed components primarily through putting in more care, thus placing limited value on the role of patients and carers as potential partners in care.^31^, ^46^, ^47^ The evidence generated within our research programme suggests that care responsibilities are ‘handed over’ to the hospital system on admission and that this continues throughout the patient stay. Hospital care tacitly encourages passivity, contributing to patients being ill‐equipped for returning and managing at home, thereby risking unplanned hospital readmission.^21^ This key finding, supported by the evidence that patients may be encouraged to be more forthcoming with staff^32^ and that patients and carers are increasingly recognized as stepping in when care systems are suboptimal,^41^, ^48^ provided us with a compelling rationale to develop an intervention to support patient involvement. Whilst a patient involvement intervention may appear less complex than the traditional multicomponent transitions interventions that dominate the field,^31^, ^46^, ^47^ we consider this approach to be truly patient‐centred and pivotal to good transitions.

Applying the O'Cathain et al.^14^ framework post hoc to our intervention development process has allowed us to report the development of our intervention in a structured and transparent format and appraise the framework to identify opportunities for enhancement. We have demonstrated how we addressed the framework principles and consider these to be a valuable reference point to support intervention development going forward. However, in our experience, two of these principles, being dynamic (constantly changing and looking forward) and iterative (taking the learning and repeating over and over), may be challenging to achieve in the context of developing *and* evaluating a complex healthcare intervention within a time‐bound study. Although we only report the preceding stages up to and including the main design phases, we have built in a subsequent pilot study,^26^ to allow for further development, and pretrial feasibility testing,^27^ which focuses primarily on developing the implementation strategy and testing out trial processes. This, at most, suggests two iterations that may not be enough. We suggest that it is not that the principles themselves are unachievable, rather that it is the *design* of health service research projects that presents the challenges. Most, if not all, are designed according to a waterfall model whereby stages are largely predefined, time‐bound, linear and sequential.^49^ Depending on the framework adopted, ‘optimization’ of the intervention may only take place within one formal iteration, with subsequent feasibility work often focusing on assessing trial processes and informing implementation plans. While the most recent MRC guidance on the development of complex interventions advocates repeating phases ‘where uncertainty remains’, there is little guidance on how this might be achieved within predefined timelines and funding constraints.^6^ Inflexibility in the funding and research process often prevents researchers from being able to truly iterate and thus truly achieve an optimal intervention that is more likely to be deliverable and effective. Adopting more agile approaches (as in software engineering) or integrating an action research phase into the development may therefore ensure greater iteration and also allow for earlier termination of projects that are unlikely to be effective (Table 7). Given the extent of research waste within health services research,^50^ a fundamental shift towards more agile approaches may therefore be required.

**Table 7:** Suggestions for enhancing the O'Cathain et al. framework (principles and actions) for complex intervention development

Original (principles and actions)	Suggestions
Iterative and dynamic	Be open to how about what is realistically achievable within a time‐bound study and/or at the time of planning, build in sufficient time and evaluative steps to ensure that uncertainty during intervention development is minimized. Constant change through being dynamic also needs to recognize time to pause and reflect.
Creative	Expand the meaning to include delving into creative practices for the evolution and testing of ideas and using novel methodologies that facilitate new ways to explore problems and solutions.
Missing action	‘Creation of functions or principles’ for the future intervention to bracket and guide intervention development. This further allows for transparent reporting of intervention ideas that were suggested but not taken forward.
Missing action	‘Consolidation of evidence’ to capture *how* the evidence from disparate sources (including the co‐design stage) is brought together.

Within the framework, further guidance in the principle of ‘being creative’ is desirable. First, we require a little more clarity on the terminology. Being creative (i.e., simply having ideas) should be inherent. We consider that adopting ‘creative practices’ whereby ideas are expressed and then ‘played’ with to evolve them is more relevant to the process of developing an intervention. Second, being creative may also be about seeking out opportunities in the form of less well‐known/novel methodologies that can be adapted to support intervention development. In our research, the application of FRAM^18^ and the way in which it was applied were novel.^19^ The FRAM allowed us to take our extensive detailed qualitative work, reorientate it back into the care system and focus on functions of care activities rather than the tasks themselves. Through this method, we were able to understand the implications of handovers of care for patients, how risks that are mitigated in hospital are shifted downstream into the patient's home and, crucially, identify the key focus of our intervention leading to the development of our initial theory of change. Being creative, therefore, is about taking risk, seeking opportunities and looking outwards to robust novel approaches that permit a fresh look at what we think we know.

Intervention development papers do not tend to report a pre‐empted intervention format; therefore, they do not have to be transparent about how researchers can demonstrate that they are open to change in this respect. We deliberately report on what the original intervention was envisaged to be and thereafter explain how and why some aspects of it remained unchanged or were different.

We identified what we consider to be two additional actions for the framework: creation of either functions or intervention principles and consolidation of evidence (Table 7). From the outset, we defined our own principles for guiding the intervention development through a set of statements that set the parameters for the intervention. We revisited and enhanced these so that they collectively became a decision‐making tool to guide the content for the intervention. We considered this to be a distinct and essential action within the research process and therefore a worthwhile addition to the framework. The evidence that we amassed during the first two work packages, the multiple stakeholder engagement approaches and the various reviews had the potential to be unwieldy. Consolidation of evidence might be regarded as one of the stages where the magic in the intervention development occurs, but more transparency is required. The use of principles and transparency about how evidence is consolidated and the intervention emerges are key tenets of intervention development.

### 3.1 Limitations

O'Cathain et al.'s^14^ framework has facilitated reflection on our intervention design process and consideration of its limitations. Only two of our four co‐design workshops actively contributed to the content and format for the intervention itself. We did supplement this with additional PPI sessions. Although more involvement from patients and staff would have been valuable, it would have been difficult to hold more of these, as each workshop required a half‐day attendance. A compromise might have been to extend the design phase, taking our intervention out to a number of wards to gather feedback and discuss implementation approaches. This would have allowed a greater opportunity for small‐scale, less burdensome (for patients and staff) iteration cycles.

We found the co‐design workshops both rewarding and challenging. For us, entering into the co‐design phase with prescribed principles and functions was essential but we recognized that these might stymy the creativity of the group. Participants did, however, make valuable contributions to both the intervention format and supported decision‐making about the content. Some format suggestions were challenging to reconcile within the scope defined by our principles, theory of change and the practical constraints of the project resources (time and budget). This is clearly an area that requires further debate; however, the sheer scale of this topic places it beyond the scope of this report. In a future article, we intend to reflect on this from a number of case studies. On balance, our principles and functions enabled us to demonstrate the provenance of the intervention, guide the participants and report a coherent and clear account of its development.

## 4 CONCLUSIONS

The new MRC guidance advocates bolder, deliberative and flexible approaches to complex healthcare intervention development and evaluation. We agree with this as long as it is aligned with funders who are fully supportive of, in particular, more flexible approaches. We consider that without transparent and full reporting of the intervention development process guided by a robust framework, as researchers, we are unlikely to meet the dual aims and responsibilities of creating useful and impactful interventions whilst reducing research waste.

## AUTHOR CONTRIBUTIONS

Jenni Murray led in the design of the work and in the acquisition, analysis and interpretation of the data. Ruth Baxter, Natasha Hardicre, Rosemary Shannon, Joe Langley, Rebecca Partridge and Jane O'Hara contributed to the design of the work and in the acquisition, analysis and interpretation of the data. Rebecca Lawton and Jane O'Hara led in the conception and design of the work. Sally Moore made substantial contributions to the acquisition, analysis and interpretation of the data.

## CONFLICT OF INTEREST

The authors declare no conflict of interest.

## Data Availability

The data that support the findings of this study are available from the corresponding author upon reasonable request.

## References

[1] Hoddinott P . A new era for intervention development studies. Pilot Feasibility Stud. 2015;1:36. 10.1186/s40814-015-0032-0 27965814 PMC5153779

[2] Craig P , Dieppe P , Macintyre S , Michie S , Nazareth I , Petticrew M . Developing and evaluating complex interventions: the new Medical Research Council guidance. BMJ. 2008;337:a1655. 18824488 10.1136/bmj.a1655 PMC2769032

[3] Meijel B , Gamel C , Swieten‐Duijfjes B , Grypdonck MHF . The development of evidence‐based nursing interventions: methodological considerations. J Adv Nurs. 2004;48(1):84‐92. 10.1111/j.1365-2648.2004.03171.x 15347414

[4] De Silva MJ , Breuer E , Lee L , et al. Theory of change: a theory‐driven approach to enhance the Medical Research Council's framework for complex interventions. Trials. 2014;15:267. 24996765 10.1186/1745-6215-15-267 PMC4227087

[5] Wight D , Wimbush E , Jepson R , Doi L . Six steps in quality intervention development (6SQuID). J Epidemiol Community Health. 2016;70:520‐525. 26573236 10.1136/jech-2015-205952 PMC4853546

[6] Skivington K , Matthews L , Simpson SA , et al. A new framework for developing and evaluating complex interventions: update of Medical Research Council guidance. BMJ. 2021;374:n2061. 10.1136/bmj.n2061Skivington 34593508 PMC8482308

[7] Bleijenberg N , de Man‐van Ginkel JM , Trappenburg JCA , et al. Increasing value and reducing waste by optimizing the development of complex interventions: enriching the development phase of the Medical Research Council (MRC) framework. Int J Nurs Stud. 2018;79:86‐93. 10.1016/j.ijnurstu.2017.12.001 29220738

[8] Clarke D , Jones F , Harris R , Robert G , Collaborative Rehabilitation Environments in Acute Stroke (CREATE) Team . What outcomes are associated with developing and implementing co‐produced interventions in acute healthcare settings? A rapid evidence synthesis. BMJ Open. 2017;7:e014650. 10.1136/bmjopen-2016-014650 PMC5734495 28701409

[9] Voorberg WH , Bekkers VJJM , Tummers LG . A systematic review of co‐creation and co‐production: embarking on the social innovation journey. Public Manag Rev. 2015;17:1333‐1357.

[10] Dalgetty R , Miller CB , Dombrowski SU . Examining the theory effectiveness hypothesis: a systematic review of systematic reviews. Br J Health Psychol. 2019;24:334‐356. 30793445 10.1111/bjhp.12356

[11] Turner KM , Rousseau N , Croot L , et al. 2019 Understanding successful development of complex health and healthcare interventions and its drivers from the perspective of developers and wider stakeholders: an international qualitative interview study. BMJ Open. 2019;9:e028756. 10.1136/bmjopen-2018-028756. PMC6549621 31152042

[12] Chalmers I , Glasziou P . Avoidable waste in the production and reporting of research evidence. Lancet. 2009;374(9683):86‐89. 19525005 10.1016/S0140-6736(09)60329-9

[13] Glasziou P , Chalmers I . Is 85% of health research really “wasted”? The BMJ Opinion; 2016. Accessed September 15, 2021. https://blogs.bmj.com/bmj/2016/01/14/paul-glasziou-and-iain-chalmers-is-85-of-health-research-really-wasted/

[14] O'Cathain A , Croot L , Duncan E , et al. Guidance on how to develop complex interventions to improve health and healthcare. BMJ Open. 2019;9:e029954. 10.1136/bmjopen-2019-029954 PMC6701588 31420394

[15] Duncan E , O'Cathain A , Rousseau N , et al. Guidance for reporting intervention development studies in health research (GUIDED): an evidence‐based consensus study. BMJ Open. 2020;10:e033516. 10.1136/bmjopen-2019-033516 PMC7245409 32273313

[16] Murray J , Hardicre N , Birks Y , O'Hara J , Lawton R . How older people enact care involvement during transition from hospital to home: a systematic review and model. Health Expect. 2019;22(5):883‐893. 10.1111/hex.12930 31301114 PMC6803411

[17] Hollnagel E , Braithwaite J , Wears RL . Resilient Health Care. Ashgate; 2013. 10.1093/intqhc/mzv063 26294709

[18] Hollnagel E . FRAM, the Functional Resonance Analysis Method: Modelling Complex Socio‐Technical Systems. Ashgate; 2012.

[19] O'Hara J , Baxter R , Hardicre N . Handing over to the patient: a FRAM analysis of transitional care combining multiple stakeholder perspectives. Appl Ergon. 2020;85:103060. 10.1016/j.apergo.2020.103060 32174348

[20] Hardicre NK , Birks Y , Murray J , et al. Partners at Care Transitions (PACT)—exploring older peoples' experiences of transitioning from hospital to home in the UK: protocol for an observation and interview study of older people and their families to understand patient experience and involvement in care at transitions. BMJ Open. 2017;7(11):e018054. 10.1136/bmjopen-2017-018054 PMC5719264 29196483

[21] Hardicre N , Murray J , Shannon R , et al. Doing involvement: a qualitative study exploring the ‘work’ of involvement enacted by older people and their carers during transition from hospital to home. Health Expect. 2021;24(6):1936‐1947. 10.1111/hex.13327 34599866 PMC8628582

[22] Baxter R , O'Hara J , Murray J , et al. Partners at Care Transitions—exploring healthcare professionals' perspectives of excellence at care transitions for older people. BMJ Open. 2018;8:e022468. 10.1136/bmjopen-2018-022468 PMC6150145 30232111

[23] Baxter R , Shannon R , Murray J , et al. Delivering exceptionally safe transitions of care to older people: a qualitative study of multidisciplinary staff perspectives. BMC Health Serv Res. 2020;20:780. 10.1186/s12913-020-05641-4 32831038 PMC7444052

[24] Oikonomou E , Chatburn E , Higham H , Murray J , Lawton R , Vincent C . Developing a measure to assess the quality of care transitions for older people. BMC Health Serv Res. 2019;19:505. 10.1186/s12913-019-4306-8 31324171 PMC6642522

[25] Oikonomou E , Page B , Lawton R , Murray J , Higham H , Vincent C . Validation of the Partners at Care Transitions Measure (PACT‐M): assessing the quality and safety of care transitions for older people in the UK. BMC Health Serv Res. 2020;20:608. https://bmchealthservres.biomedcentral.com/articles/10.1186/s12913-020-05369-1 32611336 10.1186/s12913-020-05369-1 PMC7329420

[26] Shannon R , Baxter R , Hardicre N , et al. A qualitative formative evaluation of a patient facing intervention to improve care transitions for older people moving from hospital to home. Health Expect. 2022:1‐11. 10.1111/hex.13560 PMC9700184 36056639

[27] Baxter R , Murray J , O'Hara JK , et al. Improving patient experience and safety at transitions of care through the Your Care Needs You (YCNY) intervention: a study protocol for a cluster randomised controlled feasibility trial. Pilot Feasibility Stud. 2020;6:123. 10.1186/s40814-020-00655-5 32905158 PMC7466784

[28] Langstaff C , Martin C , Brown G , et al. Enhancing community‐based rehabilitation for stroke survivors: creating a discharge link. Top Stroke Rehabil. 2014;21(6):510‐519. 25467399 10.1310/tsr2106-510

[29] Nordstrom P , Gustafson Y , Michaelsson K , Nordstrom A . Length of hospital stay after hip fracture and short term risk of death after discharge: a total cohort study in Sweden. BMJ. 2015;350:h696. 25700551 10.1136/bmj.h696 PMC4353281

[30] Schwappach D . Review: engaging patients as vigilant partners in safety: a systematic review. Med Care Res Rev. 2010;67(2):119‐148. 10.1177/1077558709342254 19671916

[31] Leppin AL , Gionfriddo MR , Kessler M , et al. Preventing 30‐day hospital readmissions: a systematic review and meta‐analysis of randomized trials. JAMA Intern Med. 2014;174(7):1095‐1107. 24820131 10.1001/jamainternmed.2014.1608 PMC4249925

[32] Burrows Walters C , Duthie E . Patients' perspectives of engagement as a safety strategy. Oncol Nurs Forum. 2017;44(6):712‐718. 10.1188/17 29052666 PMC5720142

[33] Peat M , Entwistle V , Hall J , Birks Y , Golder S . Scoping review and approach to appraisal of interventions intended to involve patients in patient safety. J Health Serv Res Policy, 15(suppl 1):17‐25. 20075123 10.1258/jhsrp.2009.009040

[34] Entwistle VA , McCaughan D , Watt IS , et al. Speaking up about safety concerns: multi‐setting qualitative study of patients' views and experiences. Qual Saf Health Care. 2010;19:e33. 10.1136/qshc.2009.039743. 21127092

[35] Davis RE , Sevdalis N , Vincent CA . Patient involvement in patient safety: how willing are patients to participate? BMJ Qual Saf. 2011;20(1):108‐114. 10.1136/bmjqs.2010.041871 21228083

[36] Mazor KM , Roblin DW , Greene SM , et al. Toward patient‐centered cancer care: patient perceptions of problematic events, impact, and response. J Clin Oncol. 2012;30:1784‐1790. 10.1200/JCO.2011.38.1384 22508828 PMC3383179

[37] Penney LS , Nahid M , Leykum LK , et al. Interventions to reduce readmissions: can complex adaptive system theory explain the heterogeneity in effectiveness? A systematic review. BMC Health Serv Res. 2018;18:894. 10.1186/s12913-018-3712-7 30477576 PMC6260570

[38] Hawe P . Lessons from complex interventions to improve health. Annu Rev Public Health. 2015;36:307‐323. 25581153 10.1146/annurev-publhealth-031912-114421

[39] May CR , Johnson M , Finch T . Implementation, context and complexity. Implement Sci. 2016;11:141. 27756414 10.1186/s13012-016-0506-3 PMC5069794

[40] Lilford RJ . Implementation science at the crossroads. BMJ Qual Saf. 2018;27:331‐332. 10.1136/bmjqs-2017-007502 PMC5867445 29183917

[41] O'Hara J , Aase K , Waring J . Scaffolding our systems? Patients and families “reaching in” as a source of healthcare resilience. BMJ Qual Saf. 2019;28:3‐6. 10.1136/bmjqs-2018-008216 29764929

[42] Fylan B , Armitage G , Naylor D , Blenkinsopp A . A qualitative study of patient involvement in medicines management after hospital discharge: an under‐recognised source of systems resilience. BMJ Qual Saf. 2018;27:539‐546. 10.1136/bmjqs-2017-006813 29146681

[43] Rainey H , Ehrich K , Mackintosh N , Sandall J . The role of patients and their relatives in ‘speaking up’ about their own safety—a qualitative study of acute illness. Health Expect. 2015;18:392‐405. 10.1111/hex.12044 23332029 PMC5060780

[44] O'Hara J , Lawton R . At a crossroads? Key challenges and future opportunities for patient involvement in patient safety. BMJ Qual Saf. 2016;25(1):565‐568. 10.1136/bmjqs-2016-005476 27334867

[45] Fisher KA , Smith KM , Gallagher TH , Huang JC , Borton JC , Mazor KM . We want to know: patient comfort speaking up about breakdowns in care and patient experience. BMJ Qual Saf. 2019;28:190‐197. 10.1136/bmjqs-2018-008159 PMC6449036 30269059

[46] Burke RE , Guo R , Prochazka AV , Misky GJ . Identifying keys to success in reducing readmissions using the ideal transitions in care framework. BMC Health Serv Res. 2014;14:423. 25244946 10.1186/1472-6963-14-423 PMC4180324

[47] Le Berre M , Maimon G , Sourial N , Guériton M , Vedel I . Impact of transitional care services for chronically ill older patients: a systematic evidence review. J Am Geriatr Soc. 2017;65:1597‐1608. 28403508 10.1111/jgs.14828

[48] Fylan B , Marques I , Ismail H , et al. Gaps, traps, bridges and props: a mixed‐methods study of resilience in the medicines management system for patients with heart failure at hospital discharge. BMJ Open. 2019;9(2):e023440. 10.1136/bmjopen-2018-023440 PMC6377507 30782879

[49] Bell TE , Thayer TA . Software requirements: are they really a problem? In: Yeh RT , Ramamoorthy CV , eds. Proceedings of the 2nd International Conference on Software Engineering. IEEE Computer Society Press; 1976:61‐68.

[50] Chalmers I , Bracken MB , Djulbegovic B , et al. How to increase value and reduce waste when research priorities are set. Lancet. 2014;383(9912):156‐165. 24411644 10.1016/S0140-6736(13)62229-1

